# Giant inframuscular lipoma disclosed 14 years after a blunt trauma: A case report

**DOI:** 10.1186/1752-1947-2-318

**Published:** 2008-09-30

**Authors:** Giuseppe Nigri, Mario Dente, Stefano Valabrega, Giacomo Beccaria, Paolo Aurello, Francesco D'Angelo, Francesco Di Marzo, Giovanni Ramacciato

**Affiliations:** 1 Department of Surgery, Sapienza University of Rome, 2nd School of Medicine, St. Andrea Hospital, Rome, Italy

## Abstract

**Introduction:**

Lipoma is the most frequent benign tumor of the soft tissue. This lesion is often asymptomatic except in cases of enormous masses compressing nervous-vascular structures. Although the diagnosis is mostly clinical, imaging tools are useful to confirm the adipose nature of the lesion and to define its anatomic border. Sometimes, lipomas may be the result of a previous trauma, such as in this patient.

**Case presentation:**

A 45-year-old man presented at our institution with a giant hard firm mass in the upper external quadrant of the right buttock disclosed after a weight loss diet. Subsequent magnetic resonance imaging showed a giant adipose mass developed beneath the large gluteal muscle and among the fibers of the medium and small gluteal muscles. When questioned on his medical history, the patient reported a blunt trauma of the lower back 14 years earlier. He underwent surgery and histological examination confirmed a giant lipoma.

**Conclusion:**

Lipomas might result from a previous trauma. It is hypothesized that the trigger mechanism is activated by cytokine and growth factors released after the trauma. We herein present an exceptional case of a giant post-traumatic lipoma which caused a painful compression on the right sciatic nerve.

## Introduction

Lipoma is the most frequent benign tumor of the soft tissue. Although lipomas have been described in almost all of the organs occurring in the central nervous system, gastrointestinal tract, muscles and joints, most of the time they arise in subcutaneous tissue and can occur in every part of the body. This lesion is often asymptomatic except in cases of enormous masses compressing nervous-vascular structures. Although the diagnosis is mostly clinical, imaging tools are useful to confirm the adipose nature of the lesion and to define its anatomic border.

Sometimes, lipomas may be the result of a previous trauma, such as in the present case. It is hypothesized that the trigger mechanism for lipoma development is activated by cytokine and growth factors released after the trauma [1]. We present a case of a giant lipoma discovered 14 years after the traumatic injury in a patient who presented with pain in the right leg.

## Case presentation

**Figure 1:**
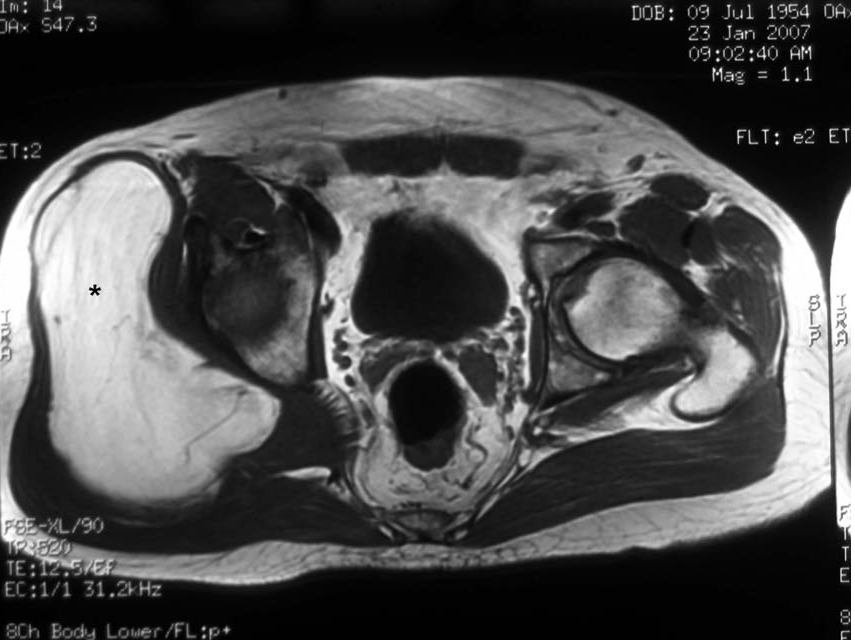
Magnetic resonance imaging showing the giant lipoma (*) underneath the right gluteus maximus.

**Figure 2:**
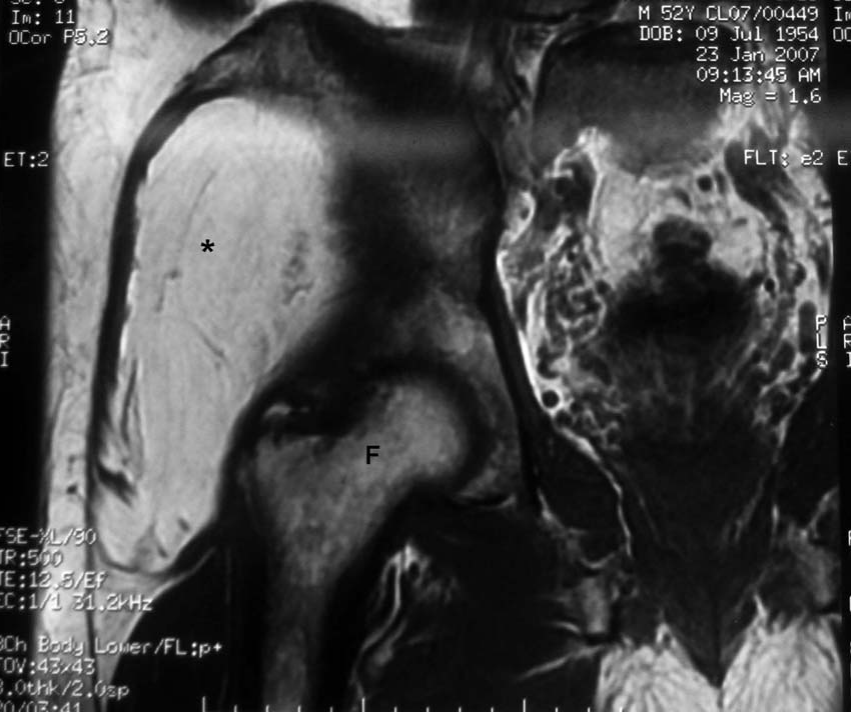
Magnetic resonance imaging (coronal view) showing the lipoma and the head of the right femur.

**Figure 3:**
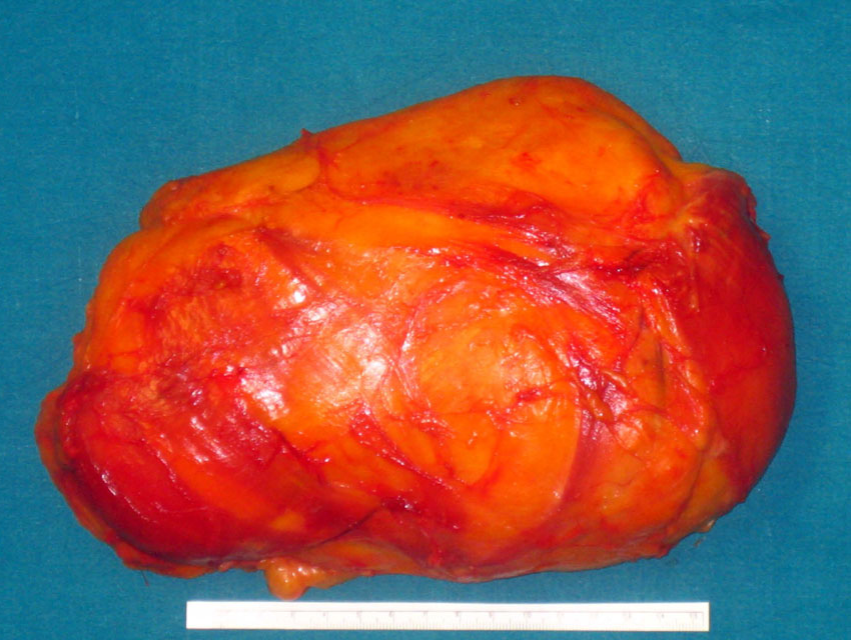
Surgical specimen (dimensions, 23 × 15 × 7 cm; weight, 1.5 kg).

A 45-year-old man presented at our institution with a giant hard firm mass in the upper external quadrant of the right buttock disclosed after a weight loss diet. His past medical history was positive for a previous blunt trauma in that area, as a result of a motor vehicle accident 14 years earlier. Since that time, he had felt a mass arising very slowly until the present time. The patient complained of pain and a functional limitation of the right leg. For this reason, he was scheduled for removal of the mass. Magnetic resonance imaging (MRI) was performed to assess the anatomy of the mass and its relationship with the surrounding structures (Figures 1 and 2). The mass was located underneath the greater gluteus muscle and among the fibers of the medial and lesser gluteus muscles. The huge mass was excised with its capsule, once the gluteus muscles were retracted apart. Its dimensions were 23 × 15 × 7 cm and its weight was 1.5 kg (Figure 3). The mass was encapsulated, presenting fibrous septa. Histologic examination showed normal adipocytes with small nuclei pushed to the periphery of the cell by a single lipid droplet. Two drains were left in place and removed on postoperative day 3 and 4, when the patient was finally discharged.

## Discussion

Lipoma represents the most frequent tumor of the soft tissues with a predilection for female, obese patients, in the 5^th ^to 7^th ^decades. Although lipomas have been described in almost all of the organs occurring in the central nervous system, gastrointestinal tract, muscles and joints, most of the time, they arise in subcutaneous tissue in every part of the body. Histologically, there are sub-types of lipomas that can be recognized by the presence of bone formations (osteolipoma), an important myxoid change (myxolipoma), rich fibrous tissue, or cartilage (chondrolipoma).

Even though there are different types of lipoma, their gross appearance does not change. However, bony areas or gray glistening formations can be found in osteolipoma and chondrolipoma variants.

The typical clinical aspect is soft, floating, lobulated and movable in the case of subcutaneous lipomas, with an average size of 3 cm. Lipomas arising from deeper compartments, such as subfascial, intramuscular or intermuscular lipomas, appear as capsulated, well circumscribed masses with smooth borders and often adherent to the muscle. They represent less frequent lipomas that can reach unusual size exceeding 10 cm, such as in this patient.

The majority of lipomas retain a stable volume or slowly increase in size, remaining painless and asymptomatic masses that rarely invade the surrounding structures. Less commonly, they reach giant volume and weight, especially in the case of post-traumatic lipomas, giving symptoms caused by nerve and vascular compression.

The reason for the uncontrolled growth is still unclear. In the last few years, various theories have been put forward such as endocrine, dysmetabolic, and genetic origin. Also, acute blunt trauma has been considered one of the possible pathogenic causes [2,3]. A recent article examined 10 patients (12 lipomas) after blunt trauma. Neither complications nor recurrences have been reported after trauma. Another article reported three cases of lipoma following trauma [4]. These lipomas arose about 6 months after the hematoma or bruise, and the treatment of choice was preventive drainage of the hematoma and compression of the site. Several other reports described the typical patient affected by lipoma as a woman who had had an acute blunt trauma to the lower back area and thigh, rich fat tissue areas in women.

The role of blunt trauma in lipoma development is primarily supported by the rearrangement of the lipomatous tissue after its transfascial herniation caused by a deep blunt injury [5]. It has been proposed that the prolapse of normal adipose tissue outside its normal borders is a trigger mechanism for lipoma development. Aust *et al. *reported 15 lipomas developed after blunt trauma [6]. It is hypothesized that the trigger mechanism is activated by cytokine and growth factors released after the trauma [6]. Signorini *et al. *supported the active role of post-traumatic released cytokine and growth factors on precursor adipose cells [7]. These factors, such as growth factor (GH) or insulin-like growth factor (IGF) should be generated by degradation products of the post-traumatic hematoma and act as a stimulus. The same study correlated the level of partial thromboplastin time (PTT) and the origin of lipomas. It was observed that PTT was elevated in seven out of 19 patients, as already shown in the literature.

## Conclusion

Lipoma represents the most frequent tumor of the soft tissues. Several authors have reported cases of post-traumatic lipoma. The mechanism that causes these lesions is not yet known. It seems to be related to the release of post-traumatic cytokine and growth factors that stimulate precursor adipose cells. Knowledge of this process would be helpful to inhibit post-traumatic lesions such as scars and lipomas, however, further studies are necessary to achieve this goal.

## Competing interests

The authors declare that they have no competing interests.

## Authors' contributions

GN designed the study, drafted and revised the manuscript. MD, SV, GB, PA, FDA, and GR carried out the data and image acquisition and participated in the writing process. All authors read and approved the final manuscript.

## Consent

Written informed consent was obtained from the patient for publication of this case report and any accompanying images. A copy of the written consent is available for review by the Editor-in-Chief of this journal.
